# Prey detection by a stepwise visual template matching mechanism

**DOI:** 10.1098/rsos.241042

**Published:** 2024-11-13

**Authors:** Jules Silverman, Brad W. Taylor

**Affiliations:** ^1^ Department of Entomology and Plant Pathology, North Carolina State University, Raleigh, NC 27695, USA; ^2^ Department of Applied Ecology, North Carolina State University, Raleigh, NC 27695, USA

**Keywords:** search image, prey switching, prey recognition, foraging theory, prey size, emergence

## Abstract

Predators can improve prey capture using a search image, and recent prey provide a visual template with which subsequent prey are compared. Considering trout feeding responses to mayfly prey of different sizes and phenological availability across years, we tested if changing relative abundances (ratios) of prey of the same species, but different body sizes, shifted trout feeding behaviour. For example, we hypothesized that a feeding switch from larger to smaller prey required continuous exposure to the novel smaller prey. The hypothesis that continuous exposure to novel small prey results in their acceptance was not supported. Rather, we discovered that trout identify novel prey using a dynamic stepwise visual neural template prey matching process, which involves the formation of focal prey template based on size or type, rejection of novel prey that do not match the size or type templates and modification of the existing or development of multiple prey templates that eventually enabled recognition of novel, small prey. We also discovered trout store multiple visual prey templates in memory. These results have implications for predator and prey dynamics, optimal foraging, the persistence of rare prey, prey species coexistence and predator selection on prey phenology.

## 1. Introduction

Optimal foraging theory and diet selection predict that an animal will choose a larger, more abundant and/or more easily captured prey item over a smaller, less abundant and/or less tractable item. Bioenergetic decisions generally underlie food selectivity by foraging predators, i.e. the metabolic cost of search, pursuit, capture and handling versus the energy rewards [1–3]. Once-abundant prey may be largely ignored in favour of new energetically valuable prey, and thus, the predator may have a stabilizing effect on animal community structure [4,5].

Predator foraging success can be enhanced by the formation of a search image; a perceptual mechanism temporarily improving the detection of cryptic prey [6,7]. The use of search images is suggested by improved prey capture success and decreased time between captures; even a single capture can improve a predator’s ability to detect prey in subsequent encounters (e.g. [8,9]). The strength of a search image may decrease with time [8], and search image formation for one type of prey may impede alternative prey detection [10]. Also, receipt of imperfect information may hinder an animal’s response to a stimulus, such that a predator may attack anything that resembles prey; the optimal criteria depend on energetic gains versus losses [6]. Furthermore, sequential priming can impact the accuracy and longevity of prey detection [11], whereby features of the last-discovered prey are remembered by the animal [12]. This recent experience, encoded in working memory, provides a template to which subsequent visual input can be compared [13].

Our knowledge of the mechanisms fish use for search image formation to optimize prey detection is limited. Learning and memory probably play a decisive role in the foraging activities of fish. Speed of attack, reaction distance and handling time of novel prey by 15-spine sticklebacks improves with experience [14]. Visual acuity in prey detection is enhanced in larger versus smaller bluegill sunfish [15], and these fish also select prey based on apparent size either due to absolute size or proximity to the fish at the instant they are detected [16]. Bamboo sharks categorize image features and types based on their overall fitness relevance [17], while colour, size, orientation and motion all facilitate efficient visual search in archerfish [18]. While each of these examples shows detailed ways in which fish detect prey, it remains unknown how fish use these in search image formation and whether the process is generalizable.

Stream-dwelling salmonids are primarily sit-and-wait predators, feeding mainly on prey drifting in the water column or on the water surface with short trips (less than 2 m) made to capture prey, and occasionally feeding from the river bottom [19]. Brown trout are visual predators selecting their food in large part from drifting and floating invertebrates [20], being prey-size selective and using a sit-and-wait capture strategy [21]. The genesis for this study was based on our observations of salmonids, mostly rainbow and brown trout, feeding on surface-drifting insects in a 50 km section of the Missouri River near Cascade Montana, USA below Holter Dam (United States Geological Survey station number 06074000). We visited the river the third week in July 2012 and the second week in July 2013. In 2012, on rough visual inspection, approximately 20% of the surface-drifting insects were mayfly subimagos (i.e. winged sexually immature adult stage unique to mayflies) of *Ephemerella excrucians*, (7–9 mm body length), and approximately 80% were mayfly imagos (i.e. winged sexually mature adult stage) of *Tricorythodes* spp. (3–5 mm body length). Both mayfly species were eaten by the resident trout. However, during July 2013, surface drift composition differed considerably from 2012. In 2013, the larger, earlier emerging, *E. excrucians* represented approximately 85% of the surface-drifting insects with later emerging *Tricorythodes* spp. representing approximately 15%. In July 2013, we visually observed trout consume *E. excrusians* and ignore *Tricorythodes* spp. subimagos. These observations indicate that in July 2012 trout had already experienced the peak of *E. excrucians* emergence and that *Tricorythodes* spp. emergence was peaking, whereas, in July 2013 trout experienced emerging *E. excrucians* but few *Tricorythodes* spp. Therefore, we hypothesized that these resident fish required experience with a critical number of the novel prey (*Tricorythodes* spp.) before switching their diet from the focal prey *E. excrusians*.

We predicted that fish would shift from the larger focal prey to the smaller novel prey of the same species when exposure to the smaller prey vastly exceeded that of the larger prey. We recognized the difficulty of testing our hypothesis of prey discrimination and switching under field conditions with ephemeral prey. Specifically, tracking and recording prey-capture events of individual fish would be difficult among dozens of conspecifics, and identifying and counting hundreds-to-thousands of minute surface-drifting mayfly adults would be impossible over time. Therefore, we developed a laboratory assay sequentially delivering different ratios of different-sized prey to individual brown trout maintained in flow-through stream tanks. This approach also allowed us to control prey size, species and movement. Our results provide new evidence for a mechanism of prey detection by visual template matching.

## 2. Methods

### 2.1. Fish collection and transport

Six male, 33–38 cm total length stream-bred brown trout, *Salmo trutta*, were collected, by angling with barbless fly, from the South Holston River tail-water, Bristol, TN, USA. Brown trout also occur in the western rivers where observations were made that stimulated our hypotheses. Fish were held within the river in mesh baskets (less than 12 h) until transport to the laboratory. Fish were transported to the laboratory in 66 l coolers (maximum of two fish per cooler) filled with river water cooled to approximately 4.4°C by adding a block of frozen river water and aerated.

### 2.2. Fish husbandry

Trout were maintained in four circulating, aerated and refrigerated (10°C) stream tanks (Living Stream, by Frigid Units, OH). The side window of each tank was covered with black cloth to reduce visibility of the experimenters and thus minimize stress on the fish. Cobble from the native stream was added as a substrate. Three fish were housed individually in 2.13 m long (530 l) experimental tanks. The remaining three fish were housed in a 2.74 m long (719 l) holding tank and separated from one another with mesh barriers. After data were collected, fish in the holding tank were swapped with fish in the experimental tanks. Tanks contained aged tap water to remove chlorine. Water quality was maintained by physical filtration (Living Stream foam insert, 1 mm nominal mesh size) and chemical (activated carbon) filtration. Forty per cent water exchanges (with aged tap water) were performed weekly. Ammonium, nitrite and nitrate levels were monitored with an API Fishcare® test kit weekly. Water was completely recirculated about every 1.5 min, and water velocity was approximately 3 cm s^−1^. An opaque overhead cover near the rear of each tank provided shade. Light from overhead fluorescent bulbs was a constant 796 lux, with a 12 h light and 12 h dark photoperiod, on at 08.00, off at 20.00. Care and use of the fish in this study followed North Carolina State University’s Institutional Animal Care and Use Committee’s (IACUC) approval protocol no. 16–090.

### 2.3. Acclimatization to experimental prey

We determined it was not feasible to field collect and adequately store without body part breakage, the extraordinarily large numbers (approx. 500 000) of fragile adult mayflies required to feed trout for the experimental period (months). Therefore, we used laboratory-reared cockroaches to establish three prey sizes (table 1). First instar *Blattella germanica*, the German cockroach, were the smallest prey, third instar *B. germanica* were medium-sized prey, adult male *B. germanica* were large-sized prey, hereafter we refer to these as small, medium and large prey (table 1). This design eliminated confounding effects of prey species identity with prey body size and provided an order of magnitude of variation in prey body size. All prey were buoyant and floated on the water surface, and all prey could be swallowed intact by the fish. Prey were killed by freezing, then thawed prior to being introduced to fish. We acclimatized the wild-caught trout to feeding in the tanks by offering earthworms approximately 5 cm in length, which were accepted after 7–14 days. Worms were subsequently withheld, and adult *B. germanica* were introduced and accepted by Day 7. The number and weight of adult *B. germanica* consumed per day (over 4 successive days) per fish until reaching satiety were mean = 195.3, 1 s.d. = 36.5, range = 125–268 individuals; mean = 10.4 g, 1 s.d. = 2.0 g, range 6.7–14.4 g. Based on this calculation, we chose a level of approximately 25% satiety (*n* = 50 adult *B. germanica,* or the equivalent weight of other prey sizes depending on the experiment).

**Table 1.** Characteristics of the prey used in the experiments. Males and females of first and third instars were physiologically uniform, as they do not develop eggs and other sex-specific differences until the adult stage. Sex ratios of first and third instar *B. germanica* were approximately 1 : 1. Adult males were used because they are more physiologically uniform than adult females. Vertical scale bars to the right of each image are 5 mm. Photographs by Heather Frantz.

	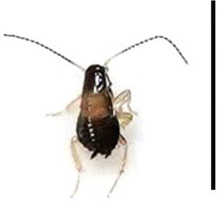	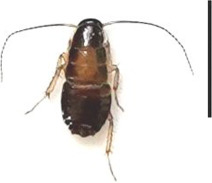	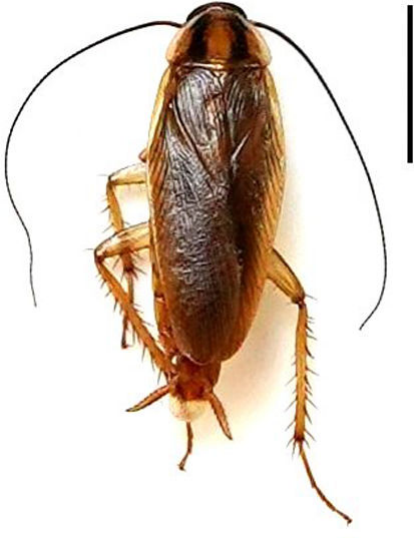	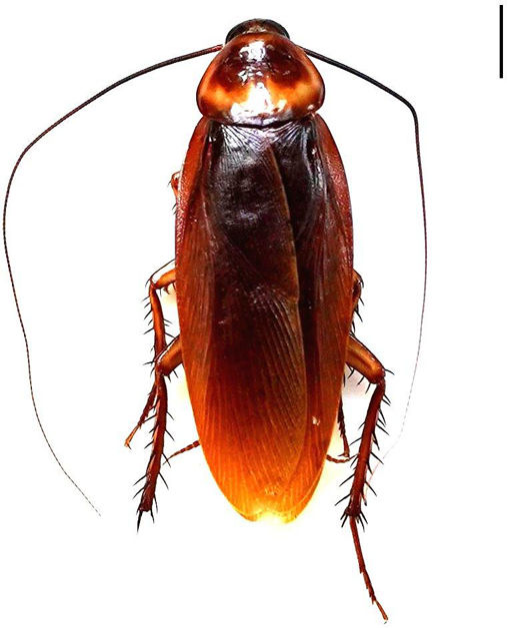
size class	small	medium	large	extra large
species	*B. germanica*	*B. germanica*	*B. germanica*	*P. americana*
developmental stage	first instar larvae	third instar larvae	adult	adult
sex	male and female	male and female	male	male
body length (mm, mean ± 1 s.e.)	2.3 ± 0.1	4.2 ± 0.1	12.5 ± 0.2	30.7 ± 0.4
Wet body weight (mg, mean ± 1 s.e.)	0.9 ± 0.1	4.3 ± 0.3	53.6 ± 0.9	784.6 ± 14.4
size class comparisons	small versus medium	medium versus large	small versus large	large versus extra-large
fractional change in length	0.79	2.0	4.3	1.5
fractional change in wet body weight	3.6	11	57	14

#### 2.3.1. Experimental procedures

Prior to introducing individual prey to a tank with a fish, an opaque cover (30 × 15 cm) was placed at an angle at the front left corner of the tank (electronic supplementary material, figure S1). This allowed prey to be dropped through an 8 cm wide slot to the water surface without the experimenter being visible to the fish. A 10 cm diameter mirror on the refrigeration unit platform at the front of the tank was angled such that we could observe the movement of prey and fish without being detected. The experiment was initiated with the fish at the back of the tank, oriented facing the current. Fish returned to this position following an encounter with prey. A prey item was dropped through the slot, and the time to consumption was recorded. The next prey was introduced after the fish returned to its resting position. Prey items drifting past the fish rested against a mesh screen and were unavailable and recorded as a rejection. All uneaten prey were removed after each daily session. We maintained a near consistent satiation level each day by adding 50 adult *B. germanica* or the equivalent weight of other prey sizes depending on the experiment.

### 2.4. Acceptance of novel prey with increasing exposure rate

We tested the prediction that novel prey would be ignored when the exposure rate, or ratio, of focal to novel prey was high, but novel prey would be consumed as their exposure rate increased, and that the effect of exposure rate would decrease as the size difference between focal and novel prey decreased.

We conducted three separate experiments, each with the same ratio of focal to novel prey but the size difference between the focal and novel prey was successively reduced. After trout satiety level was determined, fish were offered 50 adult *B. germanica* per day for 7 days, prior to beginning experiments. To investigate how the exposure rate of novel prey influenced their acceptance, trout were offered a total of 50 prey in the following ratios of focal to novel prey: 10 : 0, 9 : 1, 4 : 1, 2 : 1, 1 : 1, 1 : 2, 1 : 4, 1 : 9 and 0 : 10. For example, a daily ratio of 10 : 0 was 50 large adult *B. germanica* and 0 small first instar *B. germanica,* a ratio of 0 : 10 was 0 large adult *B. germanica* and 50 small first instar *B. germanica*, and a ratio of 1 : 1 was one large adult *B. germanica*, one small first instar *B. germanica*, etc. until 25 of each were introduced to trout. The different ratios of prey were offered to fish on successive days (e.g. Monday 10 : 0, Tuesday 9 : 1, etc.) and the proportion of novel prey always increased from 10 : 0 to 0 : 10, with daily satiety maintained by offering 50 large adult *B. germanica*, or their equivalent weight in other prey sizes depending on the experiment. All six fish received each ratio, resulting in *n* = 6 replicates per ratio.

In the first experiment comparing large-focal versus small-novel prey, we established the largest size difference between focal and novel prey using male *B. germanica* as the focal prey and the small first instar *B. germanica* as the novel prey, which produced differences of 137% in body length and 193% in wet body weight (hereafter body weight). In the second experiment with large-focal versus medium-novel prey, we reduced the size difference between the focal and novel prey by using male *B. germanica* as the focal prey (as in the first experiment) and the slighter larger third instar *B. germanica* as the novel prey, which resulted in differences of 99% in body length and 170% in body weight. In the third experiment with medium-focal versus small-novel prey, we reduced the size difference between the focal and novel prey further by using medium-sized third instar *B. germanica* as the focal prey and the small first instar *B. germanica* as the novel prey, resulting in differences of 57% in body length and 129% in body weight. We maintained consistent daily satiation levels in the experiment with the smallest difference between focal and novel prey by adding the balance of 150 third instar *B. germanica,* which were the new focal prey.

We used generalized regression models to test how feeding on novel prey changed as the ratio of novel prey increased. The response variables were per cent of novel prey consumed and time to consumption. As such, for an experiment testing large versus small prey with small prey as the novel prey and an exposure ratio of 1 : 1, if trout consumed 1 of 25 small prey then the per cent small prey consumed was 4%. Models with per cent of prey consumed as the dependent variable included a beta-binomial error distribution, and models with time to consumption included a lognormal error distribution. Individual fish were included as a random effect in all models. To test predictions about how the per cent of prey consumed or time to consumption differed among the three experiments, we used generalized linear models with experiment as the fixed effect and individual fish nested within experiment as the random effect, and error distributions were binomial for per cent consumed and lognormal for time to consumption. If the overall model was significant, then Tukey–Kramer multiple comparisons were used to assess differences among the three experiments. All statistical analyses were performed using PROC GLIMMIX and PROC FMM in SAS Studio v. 9.401M6P110718.

### 2.5. Search image extinction or prey memory

We assessed fish memory of prey using the smallest prey (first instar *B. germanica*) because they were initially rejected when offered with large-focal prey (male *B. germanica*), then accepted when offered with medium prey (see Results). Hence, we tested how long these same trout would retain their memory for small prey when deprived of this prey for 1, 2, 3, 7 and 14 days. For this experiment, we offered 50 large-focal prey (adult *B. germanica*) to each fish, individually, in sequence, immediately after the last, 0 : 10, medium versus small session in the third exposure rate experiment above. Fifty small-novel prey were then offered sequentially 24 h later (Day 0) and the time to consumption recorded. Fifty large-focal prey (male *B. germanica*) were offered immediately after this session to maintain satiety. On the following day, only the large-focal prey were offered. The next day, small-novel prey, followed by large-focal prey. This process continued providing latency periods of 0, 1, 2, 3, 7 and 14 days in small-novel prey exposure. Fifty large-focal prey were offered each day across the 14-day span to maintain satiety. The per cent of small-novel prey consumed and the time to consumption was recorded.

Generalized regression models were also used to test the prediction that the per cent of prey consumed would decrease and that the time to consumption would increase as the time since last exposure to small-novel prey (latency period) increased from 0, 1, 2, 3, 7 and 14 days for each fish. Statistical models with per cent of novel prey consumed included a beta binomial error distribution, and models with time to consumption included a lognormal error distribution. We tested for autocorrelation of the residuals using the Durbin–Watson (DW) test, and all DW values were between 1.5 and 2, indicating no first-order autocorrelation.

### 2.6. Fish response to extra-large novel prey

In addition to small-novel prey falling outside the fish’s search image due to a visual template–prey mismatch, rejection of small-novel prey by trout conditioned to large-focal prey could also be explained by small prey being undetected due to size or rejected due to foraging energetics (i.e. time, energy or both). To test the predictions that trout would not reject novel prey larger than the focal prey if acceptance was based on size or energetics alone, and there would be no change through time in the per cent consumed or time to consumption of the extra-large prey if they were within the search image template, we measured the per cent prey consumed and time to consumption by trout of prey much larger than the large-focal prey. Trout were fed 50 large-focal prey (male *B. germanica*) daily for 7 consecutive days, then on days 8–11 trout were offered large-focal prey and prey much larger than the large-focal prey, or extra-large prey, in a ratio of 9 : 1 large-focal to extra-large novel prey (*n* = 50 per day, 45 large-focal, 5 extra-large). We used male *Periplaneta americana* as the extra-large novel prey, which were identical in body form but with small differences in coloration (table 1). We analysed these data using generalized regression models identical to those used in the search image extinction and prey memory experiments above.

## 3. Results

### 3.1. Acceptance of novel prey with increasing exposure rate

#### 3.1.1. Large-focal versus small-novel prey exposure rate experiment

**Figure 1.**
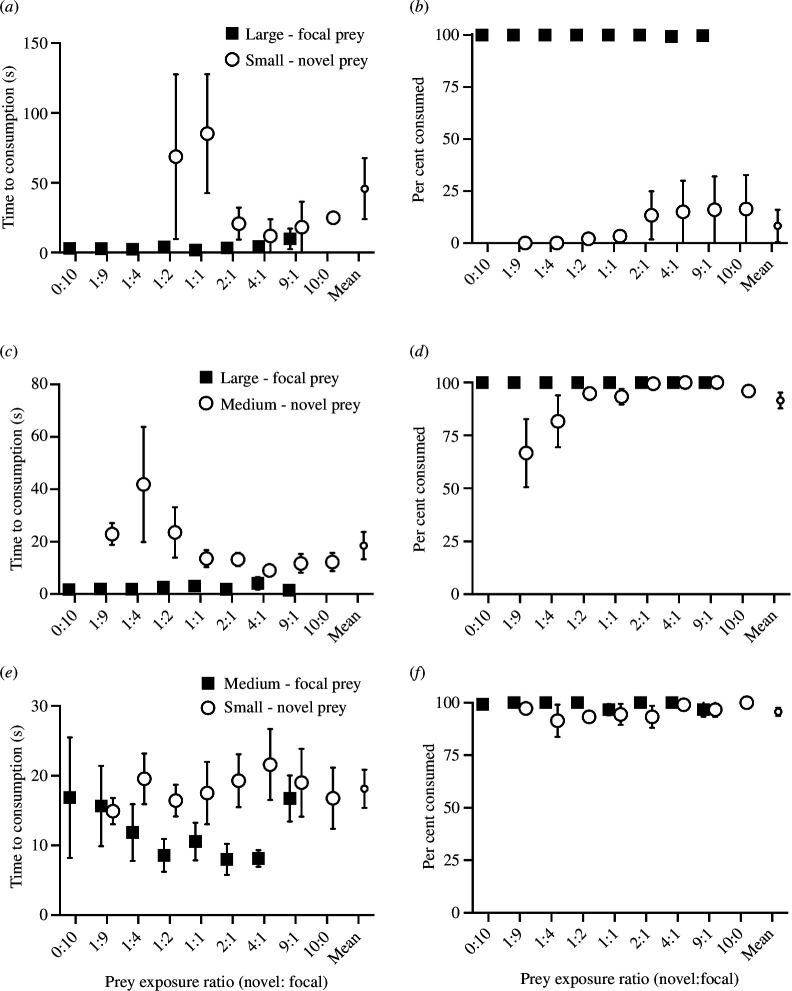
Acceptance of novel prey with increasing exposure rate or ratio. Time and per cent novel prey consumed by brown trout as the ratio of novel prey increased in three experiments in which the size differences between the focal and novel prey were progressively reduced. (*a,b*) The first experiment had the largest prey size difference, with a 137% and 193% difference in length and weight, respectively. (*c,d*) The second experiment had intermediate differences in prey size, with a 99% and 170% difference in length and weight, respectively. (*e,f*) The third experiment had the smallest prey size difference, with a 57% and 129% difference in length and weight, respectively. Note the different *y*-axis scale in panels (*a*), (*c*) and (*e*). Some error bars are obscured by the data point. The smallest point on the right of each panel is the mean across exposure ratios for the novel prey.

The time to consumption of the small-novel prey did not change as their ratio of exposure relative to large-focal prey increased (*F*_1,6_ = 3.10, *p* = 0.13, figure 1*a*). The mean time to consumption of the small-novel prey (57.2 ± 18.7 s, mean ± 1 s.e.) was 53 s longer than the time to consumption of the large-focal prey (3.9 ± 0.9 s, mean ± 1 s.e.; likelihood ratio *χ* ^2^_1,12_ = 9.14, *p* = 0.003). The per cent of the small-novel prey that were consumed also did not change as their ratio of exposure relative to the large-focal prey increased (*Z*_1,41_ = 0.19, *p* = 0.8, figure 1*b*). The mean per cent of the small-novel prey consumed (8.3 ± 3.6%, mean ± 1 s.e.) was lower than the per cent of the large-focal prey consumed (99.8 ± 0.8%, mean ± 1 s.e.; likelihood ratio *χ* ^2^_1,14_ = 10.3, *p* = 0.0014). Contrary to our prediction, increased exposure to novel prey did not stimulate greater acceptance of this novel prey item.

#### 3.1.2. Large-focal versus medium-novel prey exposure rate experiment

The time until consumption of the medium-novel prey decreased as their ratio of exposure relative to large-focal prey increased (*F*_1,41_ = 9.93, *p* = 0.003, figure 1*c*). The mean time to consumption of the medium-novel prey (18.5 ± 3.8 s, mean ± 1 s.e.) was 16.2 s longer than the time to consumption of the large-focal prey (2.3 ± 0.3 s, mean ± 1 s.e.; likelihood ratio *χ* ^2^_1,14_ = 13.13, *p* = 0.0003). However, the per cent of medium-novel prey that were consumed did not change as their ratio of exposure increased (*Z*_1,41_ = 1.70, *p* = 0.09, figure 1*d*), but there was a visible trend of more medium-sized novel prey consumed as their ratio increased. The mean per cent of medium-novel prey consumed (91.5 ± 4.1%, mean ± 1 s.e.) was not different from the per cent of large-focal prey consumed (100 ± 0%, mean ± 1 s.e.; likelihood ratio *χ* ^2^_1,14_ = 0.97, *p* = 0.3).

#### 3.1.3. Medium-focal versus small-novel prey exposure rate experiment

The time until consumption of the smallest novel prey did not change as their ratio of exposure increased (*F*_1,41_ = 0.52, *p* = 0.47, figure 1*e*). The mean time to consumption of the small-novel prey (18.1 ± 0.7 s, mean ± 1 s.e.) was 6.3 s longer than the time to consumption of the medium-prey (11.8 ± 1.2 s, mean ± 1 s.e.; likelihood ratio likelihood ratio *χ* ^2^_1,14_ = 13.99, *p* = 0.0002). The per cent of small-novel prey that were consumed also did not change as their ratio of exposure increased (*Z*_1,41_ = 0.92, *p* = 0.35, figure 1*f*). The mean per cent of small-novel prey consumed (95.7 ± 1.1%, mean ± 1 s.e.) was not different from the per cent of medium-focal prey consumed (99.1 ± 0.5%, mean ± 1 s.e.; likelihood ratio *χ* ^2^_1,14_ = 0.19, *p* = 0.6).

### 3.2. Comparison among experiments with different sizes of focal and novel prey

In the experiment with the greatest size difference between prey (large-focal versus small-novel), there was a trend for the overall time to consumption of small novel prey to be longer, but this trend was not different across the three experiments (*F*_2,10.2_ = 0.62, *p* = 0.56, figure 1*a,c,e*). However, the per cent of novel prey consumed was different (*F*_2,13.6_ = 20.2, *p* < 0.0001, figure 1*b,d,f*), with the 168% fewer novel prey consumed in the large-focal versus small-novel prey experiment compared with both the large-focal versus medium-novel prey (*t*_1,14.1_ = 5.60, *p* = 0.0002) and medium-focal versus small-novel prey (*t*_1,14.75_ = 5.53, *p* = 0.0002) experiments.

### 3.3. Search image extinction or prey memory

**Figure 2.**
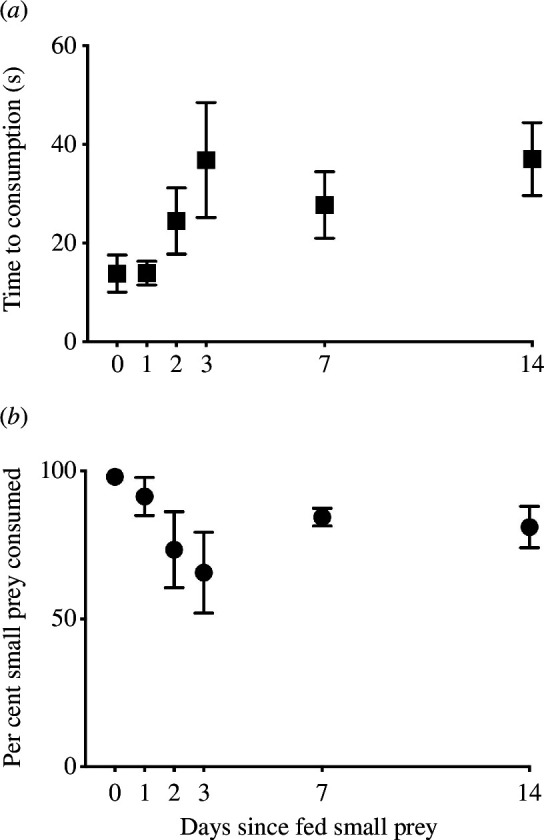
Search image or prey memory extinction. Time to consume (*a*) and per cent of small prey consumed (*b*) by brown trout after being deprived of seeing this prey for intervals of 0, 1, 2, 3, 7 and 14 days. Small prey were first instar *B. germanica*. Fifty large prey (adult *B. germanica*) were offered each day across the 14 days to maintain trout satiety.

In contrast to the large-focal versus small-novel prey experiment where nearly all small prey were not attacked, when fish were conditioned to small-novel prey in the medium-focal versus small-novel prey experiment fish consumed the small-novel prey, even when deprived of this prey for 14 days (figure 2). The time until small-novel prey were consumed increased 164% as the number of days from last exposure to this prey increased (*F*_1,29_ = 10.39, *p* = 0.003, figure 2*a*). For example, on Day 0 the time to consumption was 14 ± 4 s (mean ± 1 s.e.) but following a 14-day lack of exposure to small-novel prey, the response time was 37 ± 7 s (mean ± 1 s.e.). Despite the increase in time to consumption of small-novel prey, the per cent of small prey consumed did not decrease as the latency period of exposure to this prey increased (*Z*_1,34_ = 1.53, *p* = 0.13, figure 2*b*). Therefore, search image extinction of brown trout for this prey was at least 14 days.

### 3.4. Fish response to extra-large novel prey

**Figure 3.**
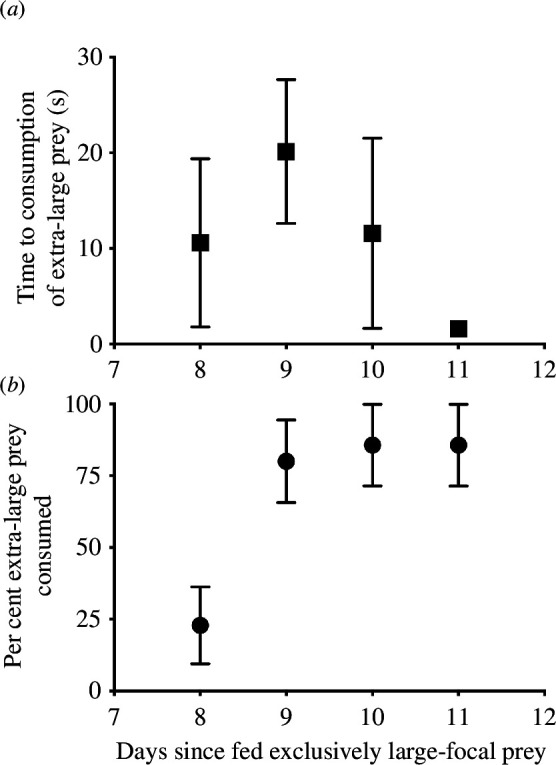
Fish response to extra-large novel prey. Time to consume (*a*) and per cent of extra-large novel prey consumed (*b*) Prey were offered in a ratio of 9 : 1 large focal to extra-large novel prey. Extra-large novel prey were male *Periplaneta americana*, which were 151% and 198% larger in length and weight, respectively, than the large-focal prey, or adult male *B. germanica*.

The time to consumption of extra-large novel prey did not change over time (time to consumption: *F*_1,22_ = 14.90, *p* = 0.0008; figure 3*a*), but the per cent of extra-large novel prey consumed did change through time, increasing from 25% to 75% over time (extra-large prey consumed: *Z*_1,22_ = 2.49, *p* = 0.01; figure 3*b*), even though one fish did not consume any of the extra large-novel prey but consumed all the large-focal prey.

## 4. Discussion

Returning to our field observation that stimulated our hypothesis and the development of the above experiments, why did Missouri River trout largely ignore *Tricorythodes* spp. in one year and attack them in a separate year? Interannual differences in timing of emergence to the adult stage by these mayflies resulted in trout experiencing different exposure rates, ratios or both between years. In 2012, our results indicate that trout developed search image templates for both mayfly prey because in July 2012 they had experienced higher exposure rates, ratios or both of the smaller *Tricorythodes* spp. In addition to the development of a search image template for the smaller *Tricorythodes* spp. in 2012, trout probably retained in memory the search image template for the earlier emerging, larger but less frequent *E. excrucians*. The recreational fly-fishing community refers to this phenomenon as pattern hangover, or fishing the false hatch, when fish retain a search image for prior prey after their availability has declined, whereas in 2013, the timing of emergence for both mayflies was delayed and by July trout had developed a search image template for only the earlier emerging, larger *E. excrucians*. According to optimal foraging theory, trout should select prey items that yield the greatest energetic gain [22]. Earlier emerging, more abundant *E. excrucians* in one year (2013) were probably the more energetically favourable mayfly prey. However, were the less abundant and smaller *Tricorythodes* spp. not detectable by these fish in July 2013? Given previous work on fish vision and body size [23], it is likely these fish could see the smaller *Tricorythodes* spp. but did not perceive them as prey either because the search image for *E. excrucians* impeded detection of these new prey [10] or because their absolute size was not deemed profitable [15]. Further, if trout did not perceive *Tricorythodes* spp. because they were novel prey with a low exposure rate and thus they fell outside the current visual prey template, then it may be an example of Weber’s Law [24]. That is, the just-noticeable difference between two stimuli could be a constant fraction (Weber fraction) of the original stimulus or, in this case, the previous focal prey. We do not know what this fraction is for these resident trout, but *Tricorythodes* spp. were approximately 50% smaller in size than *E. excrucians*; therefore, a Weber fraction of less than 1 would be predicted if these trout could not distinguish between the mayfly species. Our experimental results also showed that brown trout did not distinguish between prey sizes with a fractional difference of less than 1 (i.e. comparing body lengths of small versus medium-sized prey). However, trout did not easily distinguish between prey sizes with fractional differences of 1.5 (i.e. large versus extra large) and 2.0 (i.e. medium versus large), suggesting a Weber fraction greater than 1 for this prey body length and fish species. Learning to switch to the most visible, abundant and/or profitable prey types over time could explain frequency-dependent predation, shifts between type II and type III functional responses, coexistence of prey species [25] and be important for stabilizing community structure [26,27]. Below we discuss caveats that are important to consider in the interpretation of our results and optimal foraging decisions, search image, memory, predator satiation, and prey selection and switching within the context of our findings. We also compare our findings with other animal taxa and discuss the broader theoretical implications of our work.

The following caveats are important to consider in the interpretation of our results. Our experiments were conducted with individual trout equally well-fed surface prey, no predator threat or interspecific social interactions, constant water temperature and flow, and excellent water clarity for foraging. Hunger state and social dominance affect cognition and feeding motivation [28–30], and the threat from aerial and aquatic predators increases the energetic costs of foraging and prey selection [31], which were all controlled in this study. Habitat complexity [32] and current velocity [33] can affect the foraging efficiency of fish and can have a negative effect on trout reactive distance to prey [34,35], which were also invariant in this study. The water temperature (10°C) was within the range (7°C–12°C) where brown trout are capable of consuming and digesting at least two full meals a day [36], so biases associated with hunger or fullness were unlikely. Although these caveats can be important for the outcome of predator and prey populations, they are unlikely to alter our novel finding that trout, and potentially other predators, use a stepwise visual template matching mechanism during foraging.

**Figure 4.**
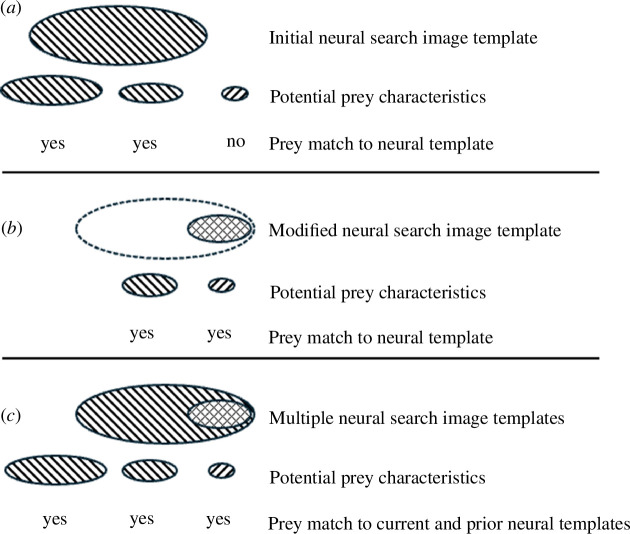
Conceptual representation of the proposed stepwise visual template matching process for prey detection. (*a*) An initial search image template in which two of the three potential prey characteristics (e.g. size or shape) are within the predator’s template but one is not. This prey detection process is represented by data in figure 1*a*–*d*. (*b*) Shows a modified search image template indicated by the cross-hatched ellipse within the initial search image template indicated by the dashed ellipse. This prey detection process is represented by the data in figure 1*e,f*. (*c*) Illustrates the use of multiple neural prey templates by predators, with at least one retained in memory, such that fish deprived of small prey retain this search image template even after repeated exposure to only large prey, as in figure 2.

Template matching, where an animal chooses a pattern whose appearance best matches a stored view, is considered one of the most basic forms of pattern vision [37,38]. Although search image formation facilitating response to novel stimuli following repeated exposure, and learning has been reported for a number of animal taxa, namely avian species, e.g. bobwhite quail, great tits, blue jays and pigeons [6,7,10,39], but also fish, e.g. sticklebacks [14]. To our knowledge, we provide the first evidence for a stepwise visual template matching process, or search image formation, whereby previously undetected prey are consumed by a predator when matched with an appropriate sized prey in the predator’s current search image template (figure 4). The steps in this process include developing an initial prey search image, or template (figure 4*a*), modifying the template (figure 4*b*) and retaining memory of prior templates while using a new or modified template (figure 4*c*). As such, the stepwise visual template matching mechanism has implications for predator and prey population dynamics, such as the persistence of rare prey that do not match a visual template and are too infrequent for the development of a template, predator and prey coexistence, and selection on prey species phenology that minimizes predation of prey of different sizes and species.

As expected based on optimal foraging theory [40], at all ratios of large-focal versus small-novel prey, the large focal prey were rapidly detected and consumed. Contrary to the prediction that encounter frequency is a prime determinant of prey selection and that trout would shift from consuming large-focal to small-novel prey as the relative availability of smaller prey exceeded larger prey, trout did not consume significantly more small prey as the ratio of small to large prey increased. As such, with the prey species and sizes and predators in this experiment, we found no or weak evidence that predators formed or improved their search image for small prey as their relative abundance increased or when small-sized prey were the only prey available (i.e. at the 10 : 0 small-novel to large-focal ratio). Further, each fish was exposed to a total of 225 small prey but on average only consumed 8.3% of small prey compared with 99.8% of 225 large prey. Although it is possible that had we continued to expose fish to only small prey that a threshold level of exposure, hunger or both (e.g. a ratio of 10 : 0 small : large prey for 3 days) may have been reached, and a higher percentage of small prey would have been consumed, as hunger and feeding motivation improved foraging efficiency of stickleback fish [28,29]. However, we decided that testing a predator’s response to a slightly larger novel prey, or medium-sized *B. germanica*, was a more novel test of the visual prey template development by predators.

Decreasing the size difference between focal and novel prey increased consumption of novel prey. As the novel prey, medium-sized prey were 57% larger in body length than the small prey and 99% smaller than the large-focal prey (adult *B. germanica*), yet when delivered sequentially with large-focal prey, the first, and all subsequent, medium-novel prey were consumed by all fish. These fish had no prior exposure to medium-sized prey, so we conclude that these prey, only slightly larger than the smallest prey, fell within a visual template or search image encoded for the large-focal prey (adult *B. germanica*) (figure 4*a*). However, was it simply an appropriate size match with the large-focal prey that triggered consumption of medium-novel prey or some other feature(s) of this prey item, such as colour, shape, pattern and movement (e.g. [18,41])? Medium-sized prey were darker and had a pattern distinct from the large-focal prey, but medium- and small-sized prey were similarly coloured and patterned (table 1) and fish consumed small-sized prey when paired with medium-sized prey, so differences in colour or pattern were unlikely explanations for consumption of the smaller, novel medium-sized prey. Also, differences in prey movement were unlikely because all prey were delivered to fish dead. Thus, it appears that decreasing the difference in prey size, or more specifically increasing the novel prey size, was key to the recognition of medium-novel prey, as we suspect this prey size now matched the trout’s initial visual prey template that was developed for the large-focal prey.

The degree of intraspecific variation in prey body size may be important for the development and flexibility of a predator’s search image template and thus predator effects on prey population dynamics. As trout’s search image expanded, or was modified to include a smaller prey (i.e. medium-sized *B. germanica*) recognition template (figure 4*b*), all small prey previously rejected were consumed when delivered sequentially, at all ratios, with a focal prey of medium size. It is also possible that the maintenance diet of medium-sized prey further enhanced small-sized prey receptivity. These data provide strong evidence for changes in the visual template by a stepwise, or at least a two-step process, which enables predators to identify prey as suitable (figure 4). Thus, body size variation in prey populations could modulate predator–prey dynamics through a visual search image neural template that could be independent of and more dynamic than traditional foraging theories based on energetics.

We hypothesized that, just as small-novel prey might not match the search image established for the large-focal prey, extra-large prey may also fall outside this visual template. Indeed, trout did not initially consume the extra-large novel prey, indicating support for the prey–template mismatch hypothesis over those based solely on size [42] or foraging energetics [40]. The rapid incorporation of extra-large novel prey into a predator’s search image template was expected given their higher probability of detection by a visual predator [6] and because many predators use foraging strategies that maximize energy gain and minimize effort or time [22,40].

Memory of prior prey may facilitate prey recognition and the development of multiple search image templates that influence predator foraging efficiency and their effects on co-occurring prey species or size classes. The fish used in medium-focal versus small-novel prey experiment had not recently (9 days) been exposed to large-focal prey. However, all large-focal prey were consumed by these fish upon reintroduction 10 days later. Similarly, small prey continued to be consumed when paired with large focal prey, demonstrating that this new expanded medium-focal versus small-novel prey visual template was stored in memory for at least 14 days. Similarly, rainbow trout can remember food items for approximately 3 months [43] and non-prey stimuli, such as a bar to trigger the release of food, are remembered for a year by goldfish [44]. Prior studies suggest that relevant learned information should be retained while information no longer eliciting a behavioural response should be forgotten [45,46]. Prey memory extinction could be related to a trade-off in the capacity to remember old prey templates and learn new ones, especially if predators experience prey that are spatially or temporally unstable [47], such as the temporal variation in emergence of different species of insects.

Our field observations of insect emergence combined with our experimental findings have implications for intra- and inter-specific prey dynamics. Differential survivorship within or among species is expected where disparate size classes (i.e. small versus large), phenotypes [48] or abundances co-occur [49,50]. In the case of intraspecific variation in body size, our findings suggest that large body size differences between overlapping cohorts could mediate overall predation on the population. Additionally, the synchronous mass emergence of insects (e.g. cicadas [51]) and, especially, mayflies [52] is widely viewed as a predator satiation strategy. Emergence patterns among species can be sequential or synchronous [52]. As such, based on our observations and experiments, we hypothesize that among species variation in the degree of synchrony, the sequence of emergence and interspecific differences in prey phenotypic characteristics (e.g. size, shape, etc.) could influence their susceptibility to predation given the importance of the stepwise visual template matching process for prey recognition. To our knowledge, the ecological and evolutionary dynamics between prey phenological patterns and predator search image development have not been explored but warrant future research given that many predator–prey interactions are affected by prey phenotype and density.

## Data Availability

The data and SAS code are available from the Dryad Digital Repository [53]. Supplementary material is available online [54].
